# Comparison of Trends in Self-reported Cigarette Consumption and Sales in England, 2011 to 2018

**DOI:** 10.1001/jamanetworkopen.2019.10161

**Published:** 2019-08-28

**Authors:** Sarah E. Jackson, Emma Beard, Bernard Kujawski, Ella Sunyer, Susan Michie, Lion Shahab, Robert West, Jamie Brown

**Affiliations:** 1 Department of Behavioural Science and Health, University College London, London, United Kingdom; 2 Sandtable, London, United Kingdom; 3 Public Health England, London, United Kingdom; 4 Department of Clinical, Educational and Health Psychology, University College London, United Kingdom

## Abstract

**Importance:**

Population cigarette consumption is declining in many countries. Accurate estimates of long- and short-term changes are vital for policy evaluation and planning. Survey data and sales data that are used to make these estimates each have important potential biases, so triangulation using different methods is required for robust estimation.

**Objectives:**

To compare monthly estimates of cigarette consumption in England from a nationally representative survey and recorded cigarette sales and to triangulate an accurate estimate of changes in cigarette consumption since 2011.

**Design, Setting, and Participants:**

This study used time series analyses based on survey data and recorded cigarette sales to estimate and compare trends in population cigarette consumption in England from 2011 to 2018. Survey participants were representative samples of 1700 people aged 16 years or older each month in England.

**Main Outcomes and Measures:**

Monthly cigarette retail sales data from August 2011 through February 2018 were obtained from a data agency. Monthly self-reports of cigarette consumption were collected over the same period using the Smoking Toolkit Study.

**Results:**

A total of 136 677 individuals (51.1% female; mean [SD] age, 46.7 [18.8] years) were surveyed. Over the study period, mean monthly cigarette consumption in England was 2.85 billion (95% CI, 2.78 billion to 2.93 billion) cigarettes based on survey data compared with 3.08 billion (95% CI, 3.03 billion to 3.13 billion) estimated from sales data. Over the whole period, cigarette consumption declined by 24.4% based on survey data and 24.1% based on sales data. This equated to 118.4 million and 117.4 million fewer cigarettes consumed per month (or approximately 1.4 billion per year) based on survey data and sales data, respectively. After adjusting for underlying trends, month-by-month changes in cigarette consumption were closely aligned: a 1% change in survey-estimated cigarette consumption was associated with a 0.98% (95% CI, 0.53%-1.44%) change in sales estimates.

**Conclusions and Relevance:**

Survey data and sales data were closely aligned in showing that overall cigarette sales in England have declined by almost a quarter since 2011, amounting to more than 1 billion fewer cigarettes smoked each year. The alignment between the 2 methods provides increased confidence in the accuracy of parameters provided by the Smoking Toolkit Study and sales data. It indicates that estimated changes in cigarette consumption are robust and provide a meaningful basis for policy evaluation and planning.

## Key Points

**Question:**

How has population cigarette consumption in England changed since 2011, and to what extent do survey measures of consumption and recorded cigarette sales produce similar estimates?

**Findings:**

This study of survey and sales data found close alignment between estimates of long- and short-term changes in population cigarette consumption in England derived from the Smoking Toolkit Study, a monthly national survey, and recorded sales. According to both methods, monthly cigarette consumption decreased by almost one-quarter between 2011 and 2018, equating to a decline of more than 117 million cigarettes per month or 1.4 billion cigarettes per year.

**Meaning:**

Cigarette consumption in England has decreased since 2011, with survey and sales data providing similar estimates.

## Teaser

This study compares estimates of changes in cigarette consumption in England between 2011 and 2018 using survey data from a nationally representative sample vs retail sales data.

## Introduction

Cigarette smoking is one of the leading risk factors for morbidity and mortality worldwide^1^ and is associated with a large economic burden.^2,3,4^ It is important to have accurate estimates of changes in aggregate cigarette consumption at a national level to evaluate and plan policies aimed at reducing smoking. These estimates can be obtained from surveys or sales data, but each has potential biases. This study compared survey and sales data in England to assess their degree of alignment and triangulate a robust estimate of national changes in cigarette consumption.

Survey data and sales data show a steady decline in smoking in many countries, including England, since the 1970s.^5,6,7^ The decline has been greater in some years than others and varies from country to country and within sociodemographic groups within countries.^5,6,8,9^ This variation has been crucial to the evaluation of policies such as tax increases, smoking bans, and marketing restrictions,^10,11,12,13,14,15^ and the rate of decline is being used in some countries to plan policies aimed at moving those countries to what has been termed the “end game” in tobacco control.^16,17,18^

National surveys provide crucial information for evaluating and planning national tobacco control policies.^19,20^ They can assess long-term progress and short-term changes that may be used to assess the effects of policies or events.^11,21,22,23^ However, they are potentially subject to biases. When it comes to estimating national cigarette consumption, measurement bias may result from not wanting to admit to smoking,^24^ inaccurate recall of number of cigarettes consumed,^25^ motivation to underestimate cigarette consumption,^26,27^ a tendency to round daily consumption to the nearest 5 or 10 cigarettes,^28^ and inaccurate translation of weight of tobacco to number of cigarettes in the case of hand-rolled cigarettes.^29^ There is evidence that failure to admit to smoking is rare in national surveys,^30^ including countries such as the United Kingdom,^31^ but it is not negligible. Sample bias may result from failure to access representative samples. This could arise from limitations in sample identification methods, inability to access certain respondents, or refusal by respondents to participate.^32^ This last issue has become increasingly challenging in recent years.^33^

Recorded sales data are potentially subject to different types of bias. These could in principle include failure of the recording method to capture the full national picture,^34^ failure to capture use of tobacco purchased illicitly or abroad,^34,35^ wastage in the sense of cigarettes bought but not consumed,^36^ and stockpiling by smokers.^37^ Recorded sales data also have the limitation that they cannot provide information on smoking prevalence, which is a crucial indicator in its own right.^5^ Also, they cannot provide a breakdown by sociodemographic characteristics.

There has been a high-profile call for science to renew its focus on triangulation.^38^ Robust estimation of changes in total national cigarette consumption requires confidence that the information being provided by survey data and sales data are in alignment. While several studies^39,40,41^ have compared survey and sales data for alcohol consumption and reported strong correlations between them, 1 study^34^ has used cigarette sales data in Scotland to estimate the sources of bias that might lead to overestimation or underestimation of consumption, and 1 study^42^ has combined sales data with smoking prevalence estimates to calculate cigarette consumption per adult smoker in Great Britain, none, to our knowledge, have examined alignment in monthly trends between cigarette survey data and sales data in England. The Smoking Toolkit Study (STS), undertaken in England, provides a unique opportunity to study this because it involves monthly surveys of representative samples in which smoking status and self-reported cigarette consumption are assessed.^43^ Previous research has shown close correspondence between major smoking indices in this survey when aggregated annually and annual national surveys.^43^

Thus, this study aimed to compare survey measures of smoking in a large sample of English adults aged 16 years and older from the STS with sales-based cigarette consumption data in England from 2011 through 2018. Specifically, this study addressed the following research questions:

1. What is the degree of concordance between estimated total national cigarette consumption in England over the study period as assessed by the STS and sales data?
2. What is the degree of concordance between the long-term trend in total national cigarette consumption in England over the study period as assessed by the STS and sales data?
3. What is the degree of concordance between monthly changes in total national cigarette consumption in England after taking account of the long-term trend and seasonal variation?

## Methods

The study used time series analyses to compare trends in population cigarette consumption in England from August 2011 through February 2018 based on survey data and recorded cigarette sales. Ethical approval for the STS was granted originally by the University College London Ethics Committee and participants provided full written informed consent. The data in this study were not collected by University College London and were anonymized before being received by University College London, thus were exempt from requiring study-specific ethical approval. This study followed the American Association for Public Opinion Research (AAPOR) reporting guideline.

### Data Sources

#### Survey Data

The STS is an ongoing monthly survey designed to provide information about smoking prevalence and behavior in England at a population level.^43^ The study uses a form of random location sampling to select a new sample of approximately 1700 adults aged 16 years or older each month. The survey typically covers 200 to 300 census output areas each wave, which are sampled at random (after stratification by geodemographic analysis of the population) from more than 170 000. Interviewers travel to the selected areas and perform computer-assisted interviews with 1 participant older than 16 years per household until quotas based on factors influencing the probability of being at home (working status, age, and sex) are fulfilled. Random location sampling is considered superior to conventional quota sampling because the choice of properties approached is reduced by the random allocation of small output areas. However, interviewers can still choose which houses within these areas are most likely to fulfil their quotas, rather than being sent to specific households in advance. Response rates are therefore not appropriate to record, unlike random probability sampling, in which interviewers have no choice as to the properties sampled, and so response at each address can be recorded. Full details of the study methods are available elsewhere,^43^ and comparisons with national data indicate that key variables such as sociodemographic characteristics and smoking prevalence are nationally representative. During the study period, data were collected from a total of 136 677 participants ranging in age from 16 to 99 years.

#### Sales Data

We obtained cigarette retail sales data from a data agency that provides measurement and analytics for market research. Data were collected from electronic point of sale (EPOS) to estimate cigarette retail sales for the Great Britain (England, Scotland, and Wales) market. The company collects census sales data from large grocery retailers on a weekly basis, comprising scanned EPOS readings of the type and volume of each pack of cigarettes sold, and a net retail price. The sampling frame includes most of the large supermarket chains but excludes discount retailers Lidl and Aldi, although neither sells cigarettes. Sales for remaining retail outlets, which are generally smaller and used for top-up and impulse purchases, are estimated using EPOS data from a weighted stratified random sample. Some independent outlets do not collect EPOS data, so sales are estimated via manual audits undertaken by the data agency, which examine invoices, purchase data, and stock levels. Data inputs are designed to be representative of Great Britain as a whole and separately for England and Wales (combined) and Scotland, and capture approximately 90% of total grocery market sales.

Sales data are not collected separately for England and Wales, but are segmented using Broadcasters’ Audience Research Board regions,^44^ with data for Wales grouped with the West of England. To get as good a match as possible between the geographical regions used in both data collection methods, we rescaled the figures for the Wales and West region to obtain reliable figures just for England. We were unable to find exact information about the geographical coverage of the West Broadcasters’ Audience Research Board region. Thus, we used the Broadcasters’ Audience Research Board map provided on the organization’s website^44^ and overlaid local authority district boundaries obtained from the UK Data Service.^45^ Based on the overlap, we identified 11 local authority districts within the West region. Next, we extracted from the Office for National Statistics (ONS) population estimate data^46^ for those identified local authority districts to estimate the number of adults (age ≥16 years, for comparability with the STS sample) in the West region (1 838 080 individuals). Finally, using the ONS 2016 figure for the Wales population aged 16 years and older (2 556 071 individuals), we calculated a scaling factor (0.42) for the West and Wales region to estimate West-only sales figures. All analyses reported here are based on our modeling of the agency-provided sales data to estimate cigarette sales in England.

### Measures

The primary measures from the STS were (1) smoking prevalence and (2) cigarette consumption among those who reported smoking. Prevalence was recorded as the proportion of participants who reported smoking cigarettes (including hand-rolled) daily or occasionally at the time of the survey or during the preceding 12 months. Smokers were identified as those who reported smoking cigarettes daily or occasionally. Self-reported consumption was measured by the question, “How many cigarettes do you usually smoke?” with participants given the choice of reporting number per day or per week, scaled up to monthly consumption by multiplying by the number of days in a given month. The mean cigarette consumption was multiplied by the smoking prevalence to give a per capita cigarette consumption figure. This was then multiplied by the total population estimate of those aged 16 years or older from the ONS for the year in question^47^ to scale up monthly consumption figures to the population in England aged 16 years or older. Where ONS population size estimates were currently unavailable (2017 and 2018 data), population size was estimated using a linear fit for the latest 6 years available in the ONS data set (2011-2016).

The primary measure from the sales data was volume sales, calculated as the number of manufactured cigarettes plus hand-rolled cigarettes estimated at 0.48 g of tobacco per cigarette.^29^

### Statistical Analysis

The analysis plan was registered on the Open Science Framework before data analysis. Data were aggregated monthly or yearly, depending on the analysis. Complete case analysis was used for the STS such that only participants with responses contributed to the aggregated level data. We analyzed data using R studio statistical software (R Project for Statistical Computing) in 3 stages.

First, we calculated total national cigarette consumption estimates from survey and sales data with the mean taken across the entire study period. Then, the 2 estimates were compared by calculating the ratio of the STS estimate over the sales estimate.

Second, we examined annual trends for cigarette consumption separately for the survey and sales data using a range of regression models and compared the overall parameters from the best-fitting models. Total national cigarette consumption from the STS and sales data were regressed on to time in separate linear regression models. Then several additional models were assessed to determine whether they provided a better fit: (1) quadratic trend model; (2) logarithmic regression (level-log model or logarithmic trend model); (3) exponential regression (log-level model or exponential trend model); and (4) power regression (log-log model or power trend model). To identify the best overall model, we compared the Akaike information criterion as the primary measure of fit and the adjusted *R*^2^ and Bayesian information criterion as secondary measures of fit. The simplest model within 2 Δ (2 Akaike information criterion units) was selected. The slopes of the 2 models were then compared by calculating the slope of the STS-time trend over the sales-time trend.

Third, we assessed the concordance between monthly estimates of cigarette consumption from the STS and sales data, using bootstrapping to calculate the confidence interval, and used autoregressive integrated moving average with exogenous input (ARIMAX) modeling.^48^ The ARIMAX method of modeling is an extension of autoregressive integrated moving average analysis, which produces forecasts based on prior values in the time series (autoregressive terms) and the errors made by previous predictions (moving average terms).

We followed a standard ARIMAX modeling approach.^48^ For this analysis, the input variable was specified as the survey-based estimate and the output variable was the sales-based estimate. First, each time series was assessed for outlying values that could bias the results; none were identified. The series were log transformed to stabilize the variance and first differenced and seasonally differenced if required. First differencing involves calculating the change between 1 observation and the next, whereas seasonal differencing involves calculating the change between 1 year and the next. The autocorrelation and partial autocorrelation functions were then examined to determine the seasonal and nonseasonal moving average and autoregressive terms. To identify the most appropriate transfer function for the continuous explanatory variables, we checked the sample cross-correlation function. We also checked that autocorrelation terms included in the model were statistically significant, and that model residuals were normally distributed, random, and independent. The Ljung-Box test for white noise was used to statistically evaluate the degree to which the residuals were free of serial correlation.

Model coefficients can be interpreted as estimates of the percentage change in sales-based total monthly national cigarette consumption for a 1% change from the series mean in the survey-based figure. All model assumptions were met.

## Results

A total of total 136 677 individuals (51.1% female; mean [SD] age, 46.7 [18.8] years) were surveyed. The mean cigarette consumption per month across the study period was 2.85 billion (95% CI, 2.78 billion to 2.93 billion) for the STS data and 3.08 billion (95% CI, 3.03 billion to 3.13 billion) for the modeled sales data. Thus the survey-based estimate was 93% of the sales estimate.

**Figure 1.**
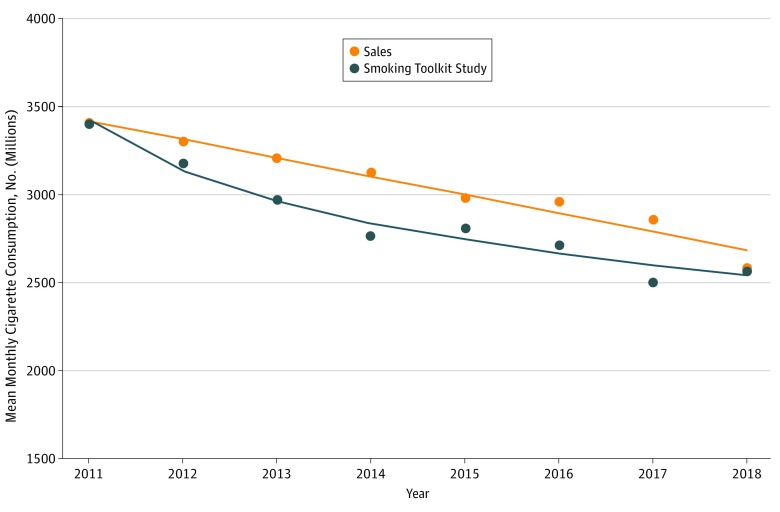
Trend Models for the Sales and Smoking Toolkit Study Data: Annualized Means of Monthly Cigarette Consumption in England From 2011 Through 2018

Figure 1 and Table 1 show the annualized means of the monthly cigarette consumption over the study period. In 2011 this was 3.40 billion according to the STS and 3.41 billion according to the sales data. In 2018 this had declined to 2.57 billion according to the STS and 2.58 billion according to the sales data. This represented an overall decline of 24.4% and 24.1% for STS and sales data, respectively, over the period. The mean monthly decline was 118.4 million according to the STS and 117.4 million according to the sales data. This amounted to 1.42 billion and 1.41 billion fewer cigarettes smoked per year according to the STS and sales data, respectively. The best-fitting curve for the annualized data was linear for the sales data but logarithmic for the STS data (Table 2).

**Table 1.** Model Fit Indices Assessing the Association Between Year and the Sales and STS Data From 2011 Through 2018

Model	Sales Data			STS Data		
	AIC	BIC	Adjusted *R*^2^^a^	AIC	BIC	Adjusted *R*^2^^a^
Linear	312.5	312.8	0.95	321.6	321.9	0.88
Quadratic	312.2	312.5	0.96	316.3	316.6	0.95
Logarithmic	322.9	323.1	0.82	314.0	314.2	0.96
Exponential	315.4	315.6	0.93	321.2	321.5	0.90
Power association	324.8	325.0	0.78	316.6	316.9	0.94

Abbreviations: AIC, Akaike information criterion; BIC, Bayesian information criterion; STS, Smoking Toolkit Study.

^a^ *R*^2^ is adjusted for the number of terms associated with each model fitted.

**Table 2.** Results of Linear Regression Analysis Assessing the Association Between Year and the Sales Data and STS Data From 2011 Through 2018

Data Source	*B* (95% CI)
STS data (log year)	−424 130 540 (−508 318 860 to −339 942 220)
Sales data (year)	−105 110 439 (−127 167 777 to −83 053 101)

Abbreviation: STS, Smoking Toolkit Study.

**Figure 2.**
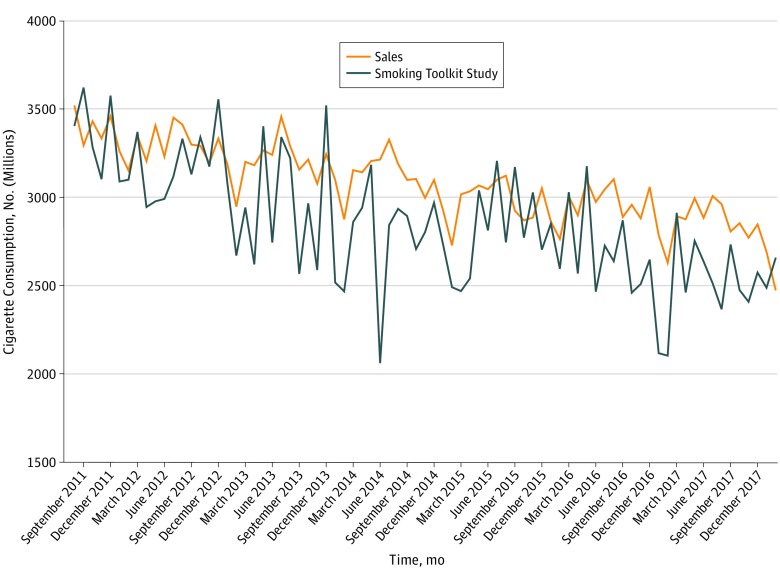
Monthly Estimates of Cigarette Consumption in England From 2011 Through 2018 Based on Self-reported Cigarette Consumption From the Smoking Toolkit Study and Recorded Sales

Monthly changes in cigarette consumption estimated by STS and sales data were highly correlated (*r* = 0.72; 95% CI, 0.57-0.82). Figure 2 shows the monthly trends. The best-fitting ARIMAX model included a nonseasonal moving average term and 1 order of differencing, and found that for every 1% decrease from the series mean in self-reported cigarette consumption, sales decreased by 0.98% (95% CI, 0.53%-1.44%). There was no evidence of outliers and all assumptions were met. However, there was some evidence of additional seasonal autocorrelation. As a sensitivity analysis, a seasonal ARIMAX model with 1 nonseasonal moving average term, a seasonal autoregressive term, and 1 order of differencing was also run. This model indicated that for every 1% decrease from the series mean in self-reported cigarette consumption, sales decreased by 0.89% (95% CI, 0.36%-1.42%). The additional seasonal term did not significantly improve the fit of the model.

## Discussion

The results showed a high degree of alignment between Smoking Toolkit Study data and sales data on the decline in aggregate cigarette consumption in England from 2011 through 2018. Both estimated that more than 1.4 billion fewer cigarettes were sold each year over the period, amounting to a decrease in consumption of approximately 24%. There was also a high correlation between the monthly changes estimated by the 2 methods after accounting for the overall trend and seasonality.

The alignment between the 2 methods provides confidence that the estimated changes in consumption are robust and provides a meaningful basis for policy evaluation and planning. The alignment also testifies to the accuracy of the component figures going into each estimate. In the case of the STS data, these figures are smoking prevalence, daily cigarette consumption among smokers, and population size. In the case of the sales data, the results validate the modeled sales figures and the extrapolation from the sample frame to retail outlets across the country.

The decline in national cigarette consumption has been dramatic and exceeded the decline in smoking prevalence, which over the same period was approximately 15%.^49^ The remainder of the decline has been in mean daily consumption. It should be noted that a decline in cigarette consumption among people who continue to smoke may not translate into a decline in toxin exposure,^50,51^ as there is evidence that smokers increase the intensity with which they smoke each cigarette to maintain their customary nicotine intake.^50,52^ However, studies have indicated that reduction may have benefits for certain disease outcomes (albeit only if the reduction is substantial and sustained),^53,54^ benefit specific population groups (eg, pregnant smokers^55,56^), and promote later attempts at cessation.^57,58,59,60^ It is also important to note that expenditure by smokers on their cigarettes has not declined because of tax increases and increases in prices charged by the tobacco companies.^61^

Although the decline in cigarette consumption was very similar in the STS and sales data, the overall level was approximately 7% lower for the STS than sales data. This may be because we overestimated the cigarette equivalence of the weight of tobacco in hand-rolled cigarettes to produce the sales-based figure. There could also have been underreporting of smoking in the survey.^24,62^ Third, the survey may have underrecruited smokers. Fourth, the survey data were restricted to people aged 16 years and older. While it is illegal in the United Kingdom for individuals younger than 18 years to purchase cigarettes, there is some smoking among those younger than 18 years.^63^

### Limitations

The study had a number of limitations. In terms of the sampling, it is possible that either or both the survey and sales data records may have failed to capture representative samples, although comparison of key variables (sociodemographic characteristics, smoking prevalence, and cigarette consumption) collected in the STS with those collected in other national surveys (the Health Survey for England and the General Lifestyle Survey) indicate that the sample is broadly representative of the adult population in England, and cigarette consumption across the surveys has been shown to be virtually identical.^43^ Over the study period, estimates of cigarette consumption among smokers participating in the STS were similar to those in the Health Survey for England^64^ and the Opinions and Lifestyle Survey (the successor to the discontinued General Lifestyle Survey)^65^ (Table 3). Thus, while both survey and sales methods may overestimate or underestimate cigarette consumption, it is unlikely results would be substantively different if these other surveys were used. In terms of measurement, the limitations of both types of data were described in the Introduction. In terms of generalizability, different countries may experience different types and degrees of bias, so one cannot assume that a similar alignment will be present in those jurisdictions. In addition, the analyses focused exclusively on cigarettes and did not include other tobacco products (eg, cigars) or alternative nicotine products (eg, electronic cigarettes), so one cannot assume that there is similar alignment for other product categories.

**Table 3.** Mean or Median Daily Cigarette Consumption Among Current Smokers in the STS, OPN, and HSE, 2011 Through 2018

Year	Cigarettes Consumed, No./d		
	STS, Mean	OPN, Mean^a^	HSE, Median^b^
2011	12.4	12.6	10
2012	12.1	11.4	10
2013	11.8	11.7	10
2014	11.4	11.1	10
2015	11.3	11.0	10
2016	11.2	11.3	10
2017	10.9	10.7	10
2018	10.6	NA	NA

Abbreviations: HSE, Health Survey for England; NA, not available; OPN, Opinions and Lifestyle Survey; STS, Smoking Toolkit Study.

^a^ Data for OPN are from the Office of National Statistics.^65^

^b^ Data for HSE are from NHS Digital.^64^

## Conclusions

This study’s findings provide the most robust quantification to date, to our knowledge, of the decline in total cigarette consumption in England at approximately 24% from 2011 through 2018. The findings also provide increased confidence in the accuracy of parameters provided by the STS methods and sales data.
